# Treatment of NAFLD with intermittent calorie restriction or low-carb high-fat diet – a randomised controlled trial

**DOI:** 10.1016/j.jhepr.2021.100256

**Published:** 2021-02-17

**Authors:** Magnus Holmer, Catarina Lindqvist, Sven Petersson, John Moshtaghi-Svensson, Veronika Tillander, Torkel B. Brismar, Hannes Hagström, Per Stål

**Affiliations:** 1 Division of Hepatology, Department of Upper GI, Karolinska University Hospital, Stockholm, Sweden; 2 Unit of Gastroenterology and Hepatology, Department of Medicine Huddinge, Karolinska Institutet, Stockholm, Sweden; 3 Medical Unit Clinical Nutrition, Karolinska University Hospital, Stockholm, Sweden; 4 Department of Clinical Science, Intervention and Technology at Karolinska Institutet, Division of Medical Imaging and Technology, Stockholm, Sweden; 5 Department of Medical Radiation Physics and Nuclear Medicine, Karolinska University Hospital, Stockholm, Sweden; 6 Godjy AB, Stockholm, Sweden; 7 Division of Clinical Chemistry, Department of Laboratory Medicine, Karolinska Institutet, Stockholm, Sweden; 8 Department of Radiology, Karolinska University Hospital in Huddinge, Stockholm, Sweden; 9 Clinical Epidemiology Unit, Department of Medicine, Solna, Karolinska Institutet, Stockholm, Sweden; 10 Department of Medicine Huddinge, Karolinska Institutet, Stockholm, Sweden

**Keywords:** Obesity, Diet treatment, Low-carb-high fat (LCHF), Intermittent calorie restriction, 5:2 diet, ALA, α-linolenic acid, ALT, alanine aminotransferase, CAP, controlled attenuation parameter, CT, computed tomography, E%, energy percent, EoT, end of treatment, HOMA-IR, homeostatic model assessment for insulin resistance, ICR, intermittent calorie restriction, IR, insulin resistance, ITT, intention-to-treat analysis, LCHF, low-carb high-fat diet, low-CHO, low-carbohydrate diet, MRS, magnetic resonance spectroscopy, MUFA, monounsaturated fatty acids, NAFLD, non-alcoholic fatty liver disease, NASH, non-alcoholic steatohepatitis, NNR, Nordic Nutrition Recommendations 2012, OGTT, oral glucose tolerance test, PP, per protocol analysis, PUFAs, polyunsaturated fatty acids, SFAs, saturated fatty acids, SoC, standard of care, T2DM, type 2 diabetes mellitus, WHR, waist-to-hip ratio

## Abstract

**Background & Aims:**

The first-line treatment for non-alcoholic fatty liver disease (NAFLD) is weight reduction. Several diets have been proposed, with various effects specifically on liver steatosis. This trial compared the effects of intermittent calorie restriction (the 5:2 diet) and a low-carb high-fat diet (LCHF) on reduction of hepatic steatosis.

**Methods:**

We conducted an open-label randomised controlled trial that included 74 patients with NAFLD randomised in a 1:1:1 ratio to 12 weeks’ treatment with either a LCHF or 5:2 diet, or general lifestyle advice from a hepatologist (standard of care; SoC). The primary outcome was reduction of hepatic steatosis as measured by magnetic resonance spectroscopy. Secondary outcomes included transient elastography, insulin resistance, blood lipids, and anthropometrics.

**Results:**

The LCHF and 5:2 diets were both superior to SoC treatment in reducing steatosis (absolute reduction: LCHF: −7.2% [95% CI = −9.3 to −5.1], 5:2: −6.1% [95% CI = −8.1 to −4.2], SoC: −3.6% [95% CI = −5.8 to −1.5]) and body weight (LCHF: −7.3 kg [95% CI = −9.6 to −5.0]; 5:2: −7.4 kg [95% CI = −8.7 to −6.0]; SoC: −2.5 kg [95% CI =−3.5 to −1.5]. There was no difference between 5:2 and LCHF (*p* = 0.41 for steatosis and 0.78 for weight). Liver stiffness improved in the 5:2 and SoC but not in the LCHF group. The 5:2 diet was associated with reduced LDL levels and was tolerated to a higher degree than LCHF.

**Conclusions:**

The LCHF and 5:2 diets were more effective in reducing steatosis and body weight in patients with NAFLD than SoC, suggesting dietary advice can be tailored to meet individual preferences.

**Lay summary:**

For a person with obesity who suffers from fatty liver, weight loss through diet can be an effective treatment to improve the condition of the liver. Many popular diets that are recommended for weight reduction, such as high-fat diets and diets based on intermittent fasting, have not had their effects on the liver directly evaluated. This study shows that both a low-carb high-fat and the 5:2 diet are effective in treating fatty liver caused by obesity.

**Clinical Trials Registration:**

This study is registered at Clinicaltrials.gov (NCT03118310).

## Highlights

• Weight reduction is the only generally available treatment for NAFLD today.
• High-fat diets, such as the LCHF, are controversial in treating NAFLD.
• The 5:2 diet has become popular and is widely used to achieve weight loss.
• In this RCT, both the LCHF and 5:2 diets were highly effective in treating NAFLD.
• The 5:2 diet reduced LDL and liver stiffness and was tolerated to a higher degree.

## Graphical abstract

**Figure:**
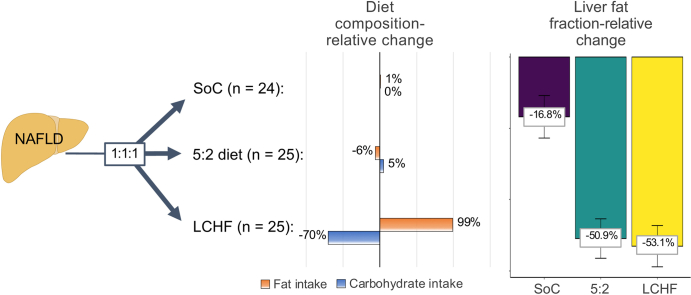

## Introduction

Non-alcoholic fatty liver disease (NAFLD) is the most common chronic liver condition, affecting approximately 25% of the world’s population.^1^ NAFLD is closely associated with obesity, type 2 diabetes mellitus (T2DM) and metabolic syndrome^2^ and has the potential for progression towards cirrhosis and liver cancer.^3^

Currently, there is no approved pharmacological treatment for NAFLD, although several compounds are under development.^4^ Instead, lifestyle changes leading to weight reduction are considered first-line treatment.^5^ Weight reduction has the potential to reverse steatosis, non-alcoholic steatohepatitis (NASH), and liver fibrosis.^6^ Some evidence suggests that diet modifications are more feasible than exercise and lifestyle changes are generally more available than bariatric surgery.^7^

It is unclear whether any specific diet should be recommended to NAFLD patients to achieve a reduction of liver fat. Recent guidelines for the treatment of NAFLD recommend a pragmatic and individualised approach and do not advocate for any specific diet. However, a macronutrient composition adjusted to the Mediterranean diet is suggested.^8^

Over the past decades, low-carbohydrate diets (low-CHO) have gained popularity. One example is the low-carb high-fat diet (LCHF diet) which has the potential for reducing body weight and insulin resistance (IR).[9], [10], [11] However, LCHF includes a high proportion of dietary fat and may increase serum levels of LDLs.^12^

Another popular diet regime is intermittent calorie restriction (ICR). One example of ICR is the 5:2 diet which is based on calorie restriction for 2 non-consecutive days per week. ICR diets may have beneficial effects on body weight and IR.[13], [14], [15] However, despite an increasing interest in ICR diets, little is known about their role as a treatment of NAFLD.^16^

Here, we present the results from a 12-week randomised controlled trial comparing treatment for NAFLD with LCHF and 5:2 diets administered by a dietitian, and standard of care (SoC) recommendations given by a physician. Magnetic resonance spectroscopy (MRS) was used to measure the reduction of liver steatosis from baseline to end of treatment (EoT).

## Materials and methods

### Study participants

Participants were recruited from 2 sources: the outpatient clinic at the Department of Hepatology, Karolinska University Hospital, Stockholm, Sweden and by advertisement in a local newspaper. Inclusion criteria were 1 of either: (1) NAFLD diagnosed by radiologic assessment (ultrasound, computed tomography [CT] or magnetic resonance imaging), (2) Fibroscan® (Echosens, Paris, France) with a controlled attenuation parameter (CAP) >280 dB/m in combination with obesity (BMI ≥30 kg/m^2^), or (3) CAP >280 and elevated alanine aminotransferase (ALT) (>46 IU/L for women, >66 IU/L for men) and overweight (BMI ≥25 kg/m^2^).

**Fig. 1:**
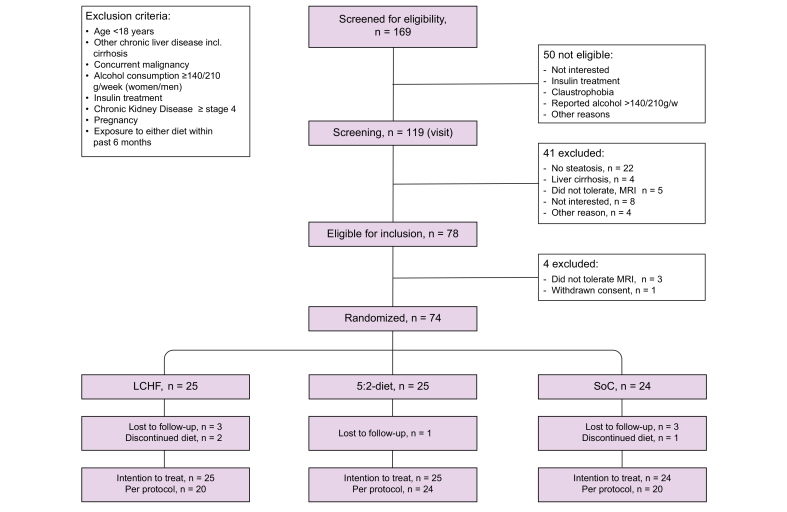
Flowchart of screening and inclusion of participants.

Exclusion criteria are listed in Figure 1. No participant was exposed to vitamin E or steatogenic medications such as steroids. At inclusion, in total 16 participants were on treatment with statins which have a potentially beneficial effect on the liver (5:2 n = 7; LCHF n = 5; SoC n = 4). All participants provided written informed consent and the study protocol was approved by the Regional Ethics Committee in Stockholm, Sweden (Dnr 2017/258-31) and registered at Clinicaltrials.gov (NCT03118310). All authors had access to the study data and reviewed and approved the final manuscript.

### Study protocol

Participants entered a 12-week intervention with either the LCHF diet, 5:2 diet, or SoC. At randomisation, a comprehensive physical assessment was undertaken (see Supplementary material). Participants were asked to quantify the level (min/week) of physical activity in a questionnaire. They were instructed not to start any dietary supplements during the intervention. At the final visit, all measures mentioned above were re-collected.

### Diet intervention

Participants were randomised in blocks of 3 in a 1:1:1 ratio by randomly drawing sealed envelopes. The allocation sequence was concealed to the participants and to all investigators. A specialist dietitian (CL) administered diet-specific advice to participants in the LCHF and 5:2 groups separately, as described below, and participants were provided written materials and diet-specific cookbooks. To support adherence to the respective diets, participants randomised to the LCHF or 5:2 diet were followed up by phone at weeks 2, 4, and 8 and a visit to the dietitian was scheduled at week 6. A detailed self-reported 3-day food diary was used to estimate the dietary intake at baseline and also to monitor adherence to diet and calculate the intake of macronutrients at week 6 and 12 of the intervention. To compare groups the intake for the 5:2 group for fasting and non-fasting days was weighted by a factor of 2 and 5, respectively, to give nutrient intake over the entire week and then reported as a daily mean. Additionally, adherence was also evaluated with a 24-h dietary recall^17^ during telephone follow up at week 2, 4, and 8 (Fig. S1).

Participants assigned to SoC treatment had an individual consultation with a hepatologist at the start of intervention. During the follow-up, they were instructed to contact the hepatologist for study-related questions by telephone or E-mail. For the SoC-group, the self-reported 3-day food diary was collected at baseline and at week 12 (Fig. S1). Thus, the SoC group served as a *placebo*-treatment and they were therefore not continuously in contact with the dietitian during the intervention.

### The 5:2 diet

On 2 non-consecutive days per week, participants in the 5:2 group were instructed to consume 500 kcal/day for women and 600 kcal/day for men. Recipes were provided with suggestions of meals that would not exceed the calorie restriction. For the remaining 5 days of the week, they received instructions and recipes that followed the *Nordic Nutrition Recommendations 2012* (NNR),^18^ with an intake limit of 2,000 kcal/day for women and 2,400 kcal/day for men. The average daily calorie intake over a week was set to 1,600 kcal/day for women and 1,900 kcal/day for men. The percentage of energy (E%) from different macronutrients in the recipes was 45–60 E% carbohydrates, 25 E% fat and 10–20 E% protein. The macronutrient composition of the NNR shares its principles with the Mediterranean diet but is adapted to local products and traditions in the Nordic countries.^18^

### The LCHF diet

Participants were instructed to consume an average daily calorie intake of 1,600 kcal/day for women and 1,900 kcal/day for men. Carbohydrate intake was restricted to a maximum of 10% of the total energy intake. Moreover, recipes were provided with an energy distribution of 5–10 E% carbohydrates, 50–80 E% fat and 15–40 E% protein. The LCHF diet is based on meat, fish, eggs, vegetables, vegetable oils, and dairy fat. Foods to be avoided included sugar, bread, pasta, rice, pies, potatoes, and fruit.

### Standard of care

The SoC group received individualised guidance from a hepatologist on how to choose a healthy diet, to reduce the intake of sweets and saturated fatty acids, increase sources of unsaturated fat, avoid large portions, and to regularly eat 3 meals per day. Each participant was provided a written summary of the dietary advice (see Supplementary material).

### Magnetic resonance spectroscopy

Twenty-four to 72 h before randomisation all participants underwent MRS.^19^ MRS data were analysed using the Java-based magnetic resonance user interface software (JAVA, Sun Microsystems, Menlo Park, California, US)^20^ and the fat and water peaks were quantified using the advanced method for an accurate and efficient spectral fitting algorithm.^21^ For a complete description of the MRS method, see the Supplementary material.

### Fatty acid composition

In addition to the self-reported data from the food diaries and 24 h recall, samples for detection of plasma total lipid fatty acid composition were collected at baseline, week 6 (for the 5:2 and LCHF group) and week 12 to estimate the dietary intake of fat as a measure of adherence to the diets (Fig. S1). The method is described in the Supplementary material.

### Statistical analysis

An *a priori* power calculation assumed a 30% relative reduction in liver fat in the LCHF and 5:2 groups *vs.* a 10% reduction in the SoC arm. With an α of 0.05 and 80% power to detect that effect, at least 16 persons per group were needed. To compensate for drop out, and to be able to detect a smaller difference in effect between the 5:2 and LCHF diets, the sample size was expanded to 25 persons per group.

The main analyses were done based on the intention-to-treat (ITT) population and missing data were assumed to be missing at random. All participants randomised to the intervention were included in the ITT analysis. To estimate pre and post values, as well as changes within and between the diets for the main outcomes, a linear mixed model was used with diet and time, including the interaction term, as fixed effects. In this analysis the participants were included as a random effect. Estimates were presented as least square means. Paired *t* tests were used to evaluate pre-post differences for all secondary outcomes and data were log-transformed when appropriate.

Two sensitivity analyses were performed. First, sex and MRS measure of liver fat fraction (MR fat percent) differed at baseline and were therefore included in the model as adjustment factors. Second, a per-protocol (PP) analysis was carried out. All participants who reported adherence to diet until EoT and who were not lost to follow-up were included in the PP analysis. R version 3.6.1 (R Foundation for Statistical Computing, Vienna, Austria) was used for all analyses.

## Results

### Participants and baseline characteristics

Between September 2017 and January 2020, a total of 74 participants were randomised to the treatment groups. See flowchart for a detailed description of the screening and inclusion of participants (Fig. 1). In total, dropouts were less common in the 5:2 arm (n = 1 [4%]) as compared with the LCHF arm (n = 5 [20%]), and SoC arm (n = 4 [17%]). Of those who discontinued the treatment, 7 were lost to follow-up. Hence, 64 participants were included in the PP analysis. One participant in the 5:2 group reported a severe adverse event during a fasting day (a fall as a result of hypoglycaemia). See the Supplementary material for reasons for discontinuation and adverse events.

At baseline, all groups were balanced on age, BMI, liver stiffness, waist-to-hip ratio (WHR), prevalence of T2DM, homeostatic model assessment for insulin resistance (HOMA-IR), blood cholesterol levels, and level of physical activity. No significant difference was observed in self-reported consumption of alcohol at baseline. The proportion of females was higher in the SoC group. Baseline self-reported energy percent (E%) intake from carbohydrates, lipids, and proteins was similar between groups. The MR fat percent at baseline was 12.0% in the 5:2 group, 12.7% in the LCHF group, and 16.6% in the SoC group (Table 1).

**Table 1:** Baseline characteristics at randomisation.

	Standard of care (n = 24)	5:2-diet (n = 25)	LCHF-diet (n = 25)
Age, years	56 (9)	57 (10)	56 (12)
Gender female, n (%)	17 (71)	12 (48)	12 (48)
Type 2 diabetes, n (%)	6 (25)	6 (24)	5 (20)
BMI, kg/m^2^	32.9 (5.2)	32.3 (2.7)	32.1 (3.8)
Body weight, kg	94.0 (18.1)	96.9 (14.3)	92.0 (11.8)
WHR	1.0 (0.1)	1.0 (0.1)	1.0 (0.1)
Systolic BP, mmHg	140 (17)	140 (15)	134 (14)
Diastolic BP, mmHg	91 (9)	89 (11)	89 (9)
MR-fat, %	16.6 (8.3)	12.0 (8.1)	12.7 (7.2)
MR-fat >5%, n (%)	23 (95.8)	21 (84.0)	21 (84.0)
Elastography, kPa	7.3 (3.0)	7.5 (3.1)	6.6 (2.8)
CAP, dB/m	339 (40)	340 (42)	330 (46)
HbA1c, mmol/L	41.8 (8.0)	42.6 (8.9)	39.8 (6.9)
HOMA-IR	8.4 (9.3)	6.8 (2.7)	6.1 (3.8)
OGTT, mmol/L	9.3 (2.0)	7.7 (2.0)	7.7 (1.6)
ALT, IU/L	76 (47)	59 (23)	59 (35)
AST, IU/L	48 (21)	37 (17)	36 (14)
Total cholesterol, mmol/L	5.3 (0.9)	5.3 (1.2)	4.8 (0.9)
LDL, mmol/L	3.2 (0.9)	3.2 (1.1)	2.9 (0.8)
HDL, mmol/L	1.3 (0.3)	1.2 (0.2)	1.1 (0.2)
Triglycerides, mmol/L	2.0 (1.4)	1.9 (0.6)	1.6 (0.7)
Total energy intake, kcal	1,897 (360)	1,900 (435)	1,890 (436)
Carbohydrate, E%	39.6 (8.3)	42.0 (6.3)	42.8 (8.2)
Protein, E%	17.5 (3.2)	16.8 (2.4)	17.9 (4.5)
Fat, E%	41.1 (6.4)	39.8 (6.0)	36.4 (7.4)
Alcohol, g/day	4.9 (8.0)	4.1 (9.6)	8.3 (12.5)
Physical activity, min/week	45.1 (23.9)	45.4 (31.0)	61.1 (44.0)

Continuous variables are presented as mean (±SD) or as number of participants (%). BP, blood pressure; CAP, controlled attenuation parameter; HOMA-IR, homeostatic model assessment for insulin resistance; MR fat, liver steatosis, %, measured with magnetic resonance spectroscopy; OGTT, oral glucose tolerance test; WHR, waist-to-hip ratio.

### Reduction of hepatic steatosis

**Fig. 2:**
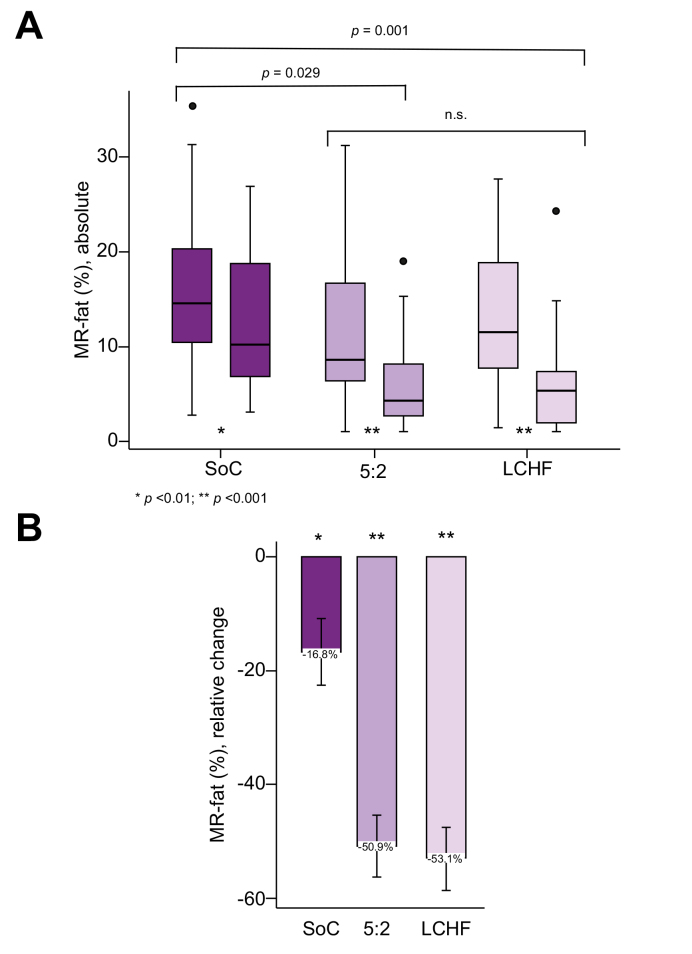
Change in liver steatosis. (A) Boxplot showing MR fat percent at baseline and at end of treatment, per group: SoC: −3.6% (95% CI = −5.8 to −1.5); 5:2: −6.1% (95% CI = −8.1 to −4.2); LCHF: −7.2% (95% CI = −9.3 to −5.1). ∗^,^∗∗*p* values for change within each group from baseline to end of treatment. *p* values at brackets, significance for between-group comparison with linear mixed model. (B) Relative change in MR fat from baseline to end of treatment, per group. ∗^,^∗∗*p* values for change within group with linear mixed model. 5:2, the 5:2 diet; LCHF, the low-carb high-fat diet; MR, magnetic resonance spectroscopy; SoC, standard of care.

In the ITT analysis, a significant reduction in liver fat was observed in all 3 groups from baseline to EoT. Absolute and relative reduction of MR fat percent is shown in Figure 2. In an inter-group comparison, the absolute reduction of MR fat percent was significantly higher in both the 5:2 and LCHF groups compared with the SoC group (difference in change 5:2 *vs.* SoC: −2.6% [95% CI = −5.0 to −0.2] and LCHF *vs.* SoC: −3.9 [95% CI = −6.3 to −1.4]). No difference in liver fat reduction was observed between the 5:2 and LCHF groups (difference in change LCHF *vs.* 5:2: −1.3 (95% CI = −3.6 to 1.1).

### Effect on liver stiffness and CAP

Liver stiffness decreased significantly from baseline to EoT in the 5:2 and the SoC group but not in the LCHF group (Table 2). In the inter-group comparison, the reduction of liver stiffness was significantly larger in both the 5:2 and the SoC group compared with the LCHF group (difference in change between 5:2 and LCHF: −1.5 kPa [95% CI = −2.5 to −0.4]) and the SoC group (difference in change between SoC and LCHF: −1.2 kPa [95% CI = −2.3 to −0.1]). CAP levels decreased from baseline in both the 5:2 and LCHF groups but not in the SoC group (Table 2). Owing to a malfunction of the Fibroscan® device (Echosens) during part of the inclusion period, the collection of CAP-levels was partly incomplete.

**Table 2:** Description of outcome measures.

	Standard of care (n = 24)			5:2-diet (n = 25)			LCHF-diet (n = 25)		
	Complete data	Δ (95% CI)	*p*	Complete data	Δ (95% CI)	*p*	Complete data	Δ (95% CI)	*p*
Weight, % reduction	21	−2.6 (−3.7 to −1.5)	**<0.001**	24	−7.4 (−8.7 to −6.2)	**<0.001**	22	−7.7 (−10.0 to −5.4)	**<0.001**
Weight, kg	21	−2.5 (−3.5 to −1.5)	**<0.001**	24	−7.4 (−8.7 to −6.0)	**<0.001**	22	−7.3 (−9.9 to −5.1)	**<0.001**
BMI, kg/m^2^	21	−0.9 (−1.3 to −0.5)	**<0.001**	24	−2.4 (−2.8 to −2.0)	**<0.001**	22	−2.5 (−3.3 to −1.7)	**<0.001**
WHR	20	−0.02 (−0.04 to −0.008)	**0.004**	24	−0.03 (−0.04 to −0.01)	**0.001**	21	−0.05 (−0.07 to −0.02)	**0.002**
Systolic BP, mmHg	20	−0.6 (−9.6 to 8.4)	0.89	24	−5.8 (−13.0 to 1.5)	0.114	22	−6.4 (−10.7 to −2.2)	**0.005**
Diastolic BP, mmHg	20	−2.1 (−6.5 to 2.2)	0.311	24	−3.8 (−7.8 to 0.3)	0.07	22	−4.0 (−7.6 to −0.3)	**0.036**
Elastography, kPa	20	−1.5 (−2.5 to −0.5)	**0.005**	24	−1.8 (−2.7 to −0.9)	**<0.001**	22	−0.3 (−1.3 to 0.7)	0.522
CAP, dB/m	13	−20.2 (−46.4 to 6.0)	0.118	16	−63.8 (−86.7 to −40.8)	**<0.001**	18	−61.9 (−84.8 to −39.0)	**<0.001**
Hba1c, mmol/mol	21	−1.1 (−2.4 to 0.2)	0.093	24	−4.8 (−6.5 to −3.0)	**<0.001**	22	−3.2 (−4.6 to −1.7)	**<0.001**
HOMA-IR	21	−2.4 (−5.2 to 0.5)	0.097	24	−3.2 (−4.1 to −2.2)	**<0.001**	22	−2.9 (−4.9 to −0.9)	**0.006**
ALT, IU/L	21	−11.8 (−17.6 to −3.5)	**0.006**	24	−17.6 (−29.4 to −11.8)	**<0.001**	22	−17.6 (−29.4 to −3.5)	**0.013**
AST, IU/L	21	−9.1 (−16.5 to −1.7)	**0.018**	24	−6.0 (−12.3 to 0.5)	0.068	22	−4.9 (−10.6 to 0.9)	0.091
Total cholesterol, mmol/L	21	−0.07 (−0.3 to 0.1)	0.452	24	−0.50 (−0.8 to −0.3)	**<0.001**	22	0.20 (−0.08 to 0.5)	0.146
LDL-cholesterol, mmol/L	20	−0.05 (−0.2 to 0.1)	0.544	24	−0.40 (−0.6 to −0.2)	**<0.001**	22	0.20 (−0.03 to 0.5)	0.075
HDL-cholesterol, mmol/L	21	0.05 (−0.01 to 0.1)	0.125	24	0.05 (−0.008 to 0.1)	0.085	22	0.10 (0.04 to 0.2)	**0.004**
Triglycerides, mmol/L	21	−0.2 (−0.4 to 0.04)	0.092	24	−0.4 (−0.6 to −0.1)	**0.004**	22	−0.2 (−0.5 to −0.01)	**0.041**
ALA + LA, mol%	18	0.4 (−2.1 to 3.0)	0.724	21	0.5 (−0.6 to 1.5)	0.374	17	1.9 (0.6 to 3.2)	**0.008**
Energy intake, kcal/day	18	−282.9 (−443.2 to −122.6)	**0.002**	23	−587.8 (−791.0 to −384.6)	**<0.001**	21	−184.1 (−383.7 to 15.5)	0.069
Carb, E%	18	−1.0 (−5.3 to 3.2)	0.614	23	1.2 (−3.2 to 5.5)	0.585	21	−30.0 (−36.1 to −24.0)	**<0.001**
Protein, E%	18	1.2 (−0.8 to 3.2)	0.237	23	2.2 (0.8 to 3.7)	**0.005**	21	3.4 (0.4 to 6.4)	**0.028**
Fat, E%	18	−0.4 (−4.5 to 3.7)	0.832	23	−3.7 (−8.6 to 1.2)	0.13	21	31.6 (26.2 to 37.0)	**<0.001**
SFA, g/day	18	−8.8 (−13.8 to −3.8)	**0.002**	23	−16.1 (−20.6 to −11.5)	**<0.001**	21	21.2 (11.8 to 30.6)	**<0.001**
PUFA, g/day	18	−0.3 (−3.8 to 3.3)	0.881	23	−2.3 (−4.9 to 0.3)	0.076	21	6.4 (2.9 to 9.8)	**0.001**
MUFA, g/day	18	−3.3 (−8.1 to 1.5)	0.164	23	−9.6 (−15.3 to −3.9)	**0.002**	21	15.6 (8.9 to 22.4)	**<0.001**
Fibre, g/day	18	0.6 (−2.8 to 4.0)	0.711	23	−4.2 (−7.6 to −0.8)	**0.018**	21	−4.8 (−7.2 to −2.4)	**<0.001**
PA, min/week	16	−3.3 (−20.7 to 14.1)	0.699	15	18.4 (−4.7 to 41.5)	0.114	14	1.6 (−35.5 to 38.7)	0.929

Change of outcome measures from baseline to end of treatment. Δ denotes relative reduction in % for weight and absolute difference of mean from baseline to end of treatment for other variables. Values of *p* denote comparisons of means at baseline and end of treatment in each group with paired *t* test. Values in bold denote statistical significance at the *p* <0.05 level.

ALA, α-linolenic acid; BP, blood pressure; CAP, controlled attenuation parameter; Carb, carbohydrate; HOMA-IR, homeostatic model assessment for insulin resistance; LA, linoleic acid; MUFA, monounsaturated fatty acids; OGTT: oral glucose tolerance test; PA, physical activity; PUFA, polyunsaturated fatty acids; SFA, saturated fatty acids; WHR, waist-to-hip ratio.

### Effect on body weight, insulin resistance, total cholesterol, and blood lipids

BMI and body weight were significantly reduced in all 3 groups, with the largest reductions observed in the 5:2 and LCHF groups. The proportions of participants who achieved weight loss of >5% and >7% was higher in the 5:2 and LCHF groups (SoC >5% n = 6 [28%], >7% n = 4 [19%]; 5:2 >5% n = 20 [83%], >7% n = 15 [63%]; LCHF >5% n = 18 [81%], >7% n = 16 [73%]). Patients in the 5:2 and LCHF groups significantly reduced HbA1c and HOMA-IR levels. A significant reduction of total serum cholesterol and LDL levels was observed in the 5:2 group but not in the LCHF or SoC groups. In fact, a trend towards higher LDL and a significantly higher HDL was observed in the LCHF group (Table 2).

### Adherence to diet

The LCHF group increased the intake of fat E% by 99% and protein E% by 24% from baseline value while carbohydrate E% decreased by 70%. In the 5:2 group protein E% increased by 16%. Intake of saturated fatty acids significantly increased in the LCHF group, but decreased in the 5:2 and SoC groups (Table 2 and Fig. S2). At the EoT, no difference was observed between the groups in the amount of consumed alcohol.

As a crude measure of total dietary fat intake, the relative levels of linoleic (LA [C18:2n-6]) and α-linolenic acid (ALA [C18:3n-6]) were increased from baseline in the LCHF group but was unchanged in the 5:2 and SoC groups (Fig. S2).

### Physical activity

Self-reported duration of physical activity did not differ between groups at baseline (Table 1). During the follow-up, no significant change in physical activity was observed for any group (Table 2).

### Sensitivity analysis

After adjusting for sex and baseline MR fat percent, no differences in our primary outcome were seen compared with the main results (Table S2). The difference in effect on liver stiffness between the LCHF and the SoC groups was no longer significant, although a trend was still observed (*p* = 0.052).

In the PP analysis the reduction of liver fat remained significant in all groups. The change in liver stiffness in the 5:2 and SoC group also remained significant (Table S3).

## Discussion

In this randomised controlled trial, we found that a dietitian-supported treatment with a LCHF or 5:2 diet was more effective than SoC in reduction of liver fat after a 12-week treatment. No difference in liver fat reduction was observed between the 5:2 and the LCHF diet. Previous studies have reported a correlation between weight loss and reduction of liver fat in NAFLD.^6^ The present results support this correlation and demonstrate that dietary advice can be individualised to achieve short-term weight loss. Individualised dietary counselling may be crucial considering that many person-specific factors affect body metabolism.^22^ The US National Institute of Health has recently announced that precision nutrition will be a major research strategy between 2020 and 2030.^23^

Although the LCHF diet has become popular over the past decade,^24^ the benefit of a low-CHO diet is controversial. Low-CHO diets, such as the LCHF, have been shown to reduce obesity and IR in patients with T2DM.^11^^,^^25^ Low-CHO diets can be equally or more effective in reducing liver steatosis in NAFLD compared with low-fat or low-calorie diets.^26^^,^^27^ However, the types of dietary lipid consumed may also be of importance in NAFLD. Previous studies have shown that obese people with a high intake of saturated fatty acids develop more steatosis compared with those who consume a diet rich in polyunsaturated fatty acids.^28^^,^^29^ Those findings were not confirmed in our study given that the LCHF diet was equally effective as the 5:2 diet in reducing steatosis in NAFLD. This finding suggests that the hypocaloric context is of greater importance when reversing hepatic steatosis than the macronutrient composition.

ICR diets, such as the 5:2 diet, have gained broad popularity and are promoted in general media.^30^^,^^31^ ICR diets have shown good results in reducing body weight, cardiovascular risk factors, and IR in obese patients.^32^ To our knowledge, only 2 studies have investigated the efficacy of ICR as a treatment for NAFLD, with both indicating that ICR can have beneficial effects on liver steatosis.^33^^,^^34^ In one of these the studies^33^ an 8-week intervention with ICR resulted in the reduction of steatosis and fibrosis as measured by ultrasound and elastography. To our knowledge, no previous study has compared the efficacy of ICR or, more specifically, the 5:2 diet with the LCHF diet to treat NAFLD.

The reduction of liver fibrosis is a key target of treatment.^3^ In this study, a significant reduction of liver stiffness was observed in the 5:2 and the SoC groups but not in the LCHF group. Although this observation is noteworthy, we cannot conclude from these results that improved liver stiffness directly translates to a reduction of liver fibrosis. Other causes for changes in elastography measurements, that is the degree of inflammation, cannot be excluded as liver biopsies were not obtained in this study.

A strong correlation exists between LDL levels and the risk of cardiovascular disease.^35^ In this trial, the 5:2 diet resulted in a reduction of LDL levels, whereas in the LCHF group a trend towards higher LDL was noted. This finding is in line with the results of other trials investigating the effect of LCHF on LDLs.^25^ NAFLD patients often have other risk factors of cardiovascular disease. Longitudinal studies of the natural course of NAFLD have shown that cardiovascular disease is the major cause of death.[36], [37], [38] Given these circumstances, LCHF could be less suited as a treatment for NAFLD patients with cardiovascular comorbidities. However, the present trial was not designed to study blood cholesterol levels specifically, and we also found a significant increase in blood HDL, known to be cardioprotective, in the LCHF arm. Therefore further research is needed to explore this issue.

Participants in the LCHF group reported more adverse events, mainly gastrointestinal, leading to diet discontinuation. As expected, patients following the 5:2 diet reported hunger and fatigue during fasting days, but few other unforeseen side effects. Fewer participants dropped out of the 5:2 group compared with the LCHF and SoC groups. This could suggest that the 5:2 diet is easier to adhere to and has less side effects, but that would need to be tested in additional studies.

This study has several strengths. First, it is a prospective randomised controlled trial with well-balanced groups at baseline. Second, a large proportion of the participants completed the study protocol. Third, records of food intake and changes in plasma lipid concentrations suggest good adherence to the diets.^39^ The outcome measure in our trial, MRS, is a well-validated and reliable non-invasive method for measuring liver steatosis.^40^ A few patients had between 3% and 5% hepatic steatosis. Previous studies have however shown that a cut-off level of 3% can be used for detecting steatosis with MRS, which is why we chose to also include patients with an MR fat percent of 3–5% at baseline.^41^ The study population is representative of the majority of NAFLD patients, ensuring this study has high external validity and suggests that these diets are appropriate to use at primary care level.

Some limitations should be acknowledged. As this trial was investigating dietary treatment of NAFLD it was designed as an open-label study. In an ideally designed randomised controlled trial the assigned treatment is double-blinded. However, this cannot be accomplished in diet interventions where the exposure is obvious to both the participants and the researchers. The duration of the trial was short (12 weeks). Short-term weight loss can be difficult to maintain over time.^42^ Our study cannot predict the long-term risk of steatosis recurrence after the intervention. To study this issue future studies should have longer follow-up periods. Despite randomisation, the SoC group was not balanced to the same extent as the 5:2 and LCHF groups for sex and baseline MRS fat percent. However, a sensitivity analysis adjusting for these factors confirmed the main results.

A 3-day food diary was used to evaluate the diet composition during the trial. When writing a food diary, patients often under-report the total amount of energy.^43^ Because of this liability, the results indicating that all 3 groups had a hypocaloric diet already at baseline should be interpreted with caution.

Finally, the SoC group did not receive the same guidance by a dietitian as the LCHF and 5:2 groups. The differences observed in the LCHF and 5:2 groups compared with the SoC group could be attributable to more frequent consultations during follow-up. However, the primary aim of this trial was to compare the effects of a low-CHO diet and ICR diet with standard of care. The objective of the design of this trial was that the SoC group served as a *placebo*-treatment. Future studies should evaluate whether the effects of the LCHF and 5:2 diets can be achieved without comprehensive guidance from a dietitian.

## Conclusions

The 5:2 and LCHF diets were equally effective in reducing liver steatosis, body weight, and measures of IR in NAFLD, supporting individual decision-making for patients with NAFLD who want to reduce hepatic steatosis. However, the 5:2 diet also reduced liver stiffness, was tolerated to a higher degree, and had more favourable effects on LDL cholesterol than the LCHF diet. Given this, the 5:2 diet could possibly be overall more beneficial to patients with NAFLD, especially to those with cardiovascular risk factors.

## Financial support

The study was supported by grants from the 10.13039/501100004348Stockholm County Council (ALF LS 2019-0064), the Dietary Science Foundation (Kostfonden), the Skandia Research Foundation and the Åke Wiberg Foundation. PS was supported by grants from the 10.13039/501100004348Stockholm County Council, Sweden (Clinical research appointment K2017-4579), the 10.13039/100012306Center for innovative medicine, Stockholm, Sweden (CIMED 20180889) and The Swedish Cancer Society (170690).

## Authors’ contributions

Study conception and design: H.H., M.H., P.S. Acquisition of data: M.H., C.L., S.P., T.B., H.H., V.T., P.S. Statistical analysis: J.M.-S., M.H. Analysis and interpretation of data: all authors. Drafting of manuscript: M.H. Critical revision: all authors. Guarantors of article: M.H., P.S. Approval of the final version of the article, including the authorship list: all authors.

## Data availability

The raw data supporting the findings of this trial was generated at Karolinska Institutet, Stockholm, Sweden. Anonymised data in the study are available from the corresponding author on reasonable request. This excludes sharing of data that is in conflict with the statement of confidentiality in the informed consent.

## Conflict of interest

The authors declare no conflicts of interest that pertain to this work.

Please refer to the accompanying ICMJE disclosure forms for further details.
