# Supplementary material for: Treatment of NAFLD with intermittent calorie restriction or low-carb high-fat diet – a randomised controlled trial
Source: JHEP Rep. 2021 Feb 17;3(3):100256. doi: 10.1016/j.jhepr.2021.100256 (PMC8059083; doi:10.1016/j.jhepr.2021.100256)
Supplement: Multimedia component 1 [file mmc1.pdf]

Treatment of NAFLD with intermittent calorie restriction or low-carb high-fat diet – a randomised controlled trial

Magnus Holmer, Catarina Lindqvist, Sven Petersson, John Moshtaghi-Svensson, Veronika Tillander, Torkel B. Brismar, Hannes Hagström, Per Stål

Table of contents

Supplementary methods..2

Table S1..7

Table S2..8

Table S3..9

Fig. S1..10

Fig. S2..11

Fig. S3..12

Supplementary references...13

Supplementary methods

Collection of data at randomization and at end of treatment

Anthropometric measures were collected. Height was measured to the nearest 0.5 cm using a standard wall-mounted stadiometer. Bodyweight was measured to the nearest 0.1 kg using a calibrated clinical scale. Waist and hip circumference were measured to the nearest millimetre by standard procedures using a 150-cm measuring tape. BMI was calculated as weight (kg)/height squared (m). Routine blood samples were drawn. The Homeostatic Model Assessment for Insulin Resistance (HOMA-IR) was calculated as fasting plasma insulin (mU/L)*fasting plasma glucose (mg/dL).[1] Fibroscan® elastography with CAP was measured. Participants without T2DM underwent an oral glucose tolerance test (OGTT). The alcohol use disorders identification test (AUDIT)[2] and blood phosphatidyl ethanol[3] were used to screen for alcohol-related liver disease.

The data from the self-reported 3-day food diary was used to calculate the daily intake of energy and selected nutrients using Dietist Net Pro-software (Kost och Näringsdata, Stockholm, Sweden). With this software, detailed estimates of the dietary composition can be extracted which are based on food composition tables from the Swedish National Food Administration. If the food item were not in the central database, information was collected from the DABAS database that collects data from food manufacturers and suppliers in Sweden.[4]

Method for analysis of plasma fatty acid composition

Measurement of the total fatty acid composition of plasma lipids was performed as follows: Lipids from 200 μL plasma were extracted using an acidic Folch-extraction[5] (120mM HCl in 2:1 Chloroform: MeOH solution) in an ultra-sonication water bath. An internal standard 196 μg/mL tricosanoic acid (C:23 FA, T6543MG Sigma) was used. The lower organic phase was collected and evaporated with N_2 and the extracted lipids were hydrolysed and methylated for 2 h at 60°C using 1.25M HCl in MeOH (Sigma). Fatty acid methyl esters (FAMEs) were then extracted using hexane and, after evaporation, dissolved in 200 μL hexane for separation and detection using an SP-2560 column, 75 m x 0,18 mm (Supelco, Merck) in an Agilent Technologies 7890B GC-FID system programmed for a temperature elevation from 100 to 240°C. FAME peak areas were determined using Agilent ChemStation software. Fatty acid composition is presented as mol-% of total fatty acids quantified.

Method of magnetic resonance spectroscopy

The MRS examinations were performed on a 3T magnetic resonance scanner (Ingenia; Philips Healthcare, Best, The Netherlands). Single voxel MRS was performed in the right hepatic lobe avoiding large blood vessels and bile ducts. A ^{1}H MRS PRESS sequence with a voxel size of 30 x 30 x 30 mm, 16 averages in 2 breath holds, echo time of 35ms and repetition time of 2000 ms. The placement of the voxel was saved and used as a reference for the 3-month follow-up.

Translation from Swedish of written instructions given to patients randomized to the standard of care arm

Advice on diet and physical activity for NAFLD

The accumulation of fat in the liver (steatosis) can be reduced through diet and exercise. For instance, it is known that sugar in sweetened beverages and juices can be stored in the liver as fat and that fat can be burned by doing regular exercise. You should try to lose weight. A weight reduction of approximately 10% can lead to the resolution of your steatosis.

You have been diagnosed with fatty liver and therefore you should observe the following list of advice:

- Avoid large servings of food.
- Try to eat slowly. Your meal should take about 15-20 minutes to eat. Only then will you have a chance to feel full after eating a normal-sized serving.
- Don't wait too long between meals but continue with scheduled meal times. It could lead to an increased feeling of hunger during the day. Then you might overeat later in the evening. Try to eat at least three meals per day.
- Avoid foods with added sugars, such as cookies, ice cream, pastries, candy, sweetened beverages and fruit juice. Good alternatives are mineral water, diet soda (with zero sugar), dark chocolate (>70% cocoa) or fresh fruits and berries. Berries contain less sugar and are a better choice than bananas, pineapples or grapes.
- Snacks, such as potato chips, popcorn and salted nuts, contain a lot of calories and should be avoided.

- Try to eat vegetables with every meal. A target can be to fill at least half your plate with vegetables. Peas, beans, broccoli, carrots and cabbage contain high amounts of dietary fibre and are preferred over lettuce, cucumber or tomato, which are low in fibre.
- Try to eat at least three servings of salmon, herring or mackerel per week.
- During the time you are in the study you should avoid drinking alcohol. Apart from that, it could harm your liver because alcohol contains a large amount of calories/energy but no real nourishment.
- Walk at least 30 minutes per day to burn calories.

Results

Table S1. Reported adverse events and reasons for lost to follow-up

Diet	Reported event	Discontinued diet	Lost to follow up
5:2	Hypoglycaemia, pre-syncope	No	No
5:2	Personal reasons	Yes	Yes
LCHF	Dyspepsia, nausea	Yes	No
LCHF	Nausea, weight gain	Yes	Yes
LCHF	Dyspepsia. Found diet difficult to implement	Yes	Yes
LCHF	Found diet difficult to implement	Yes	Yes
LCHF	Nausea, head ache.	Yes	No
LCHF	Vertigo, hypotension	No	No
SoC	Personal reasons	Yes	Yes
SoC	Depression and burn-out	Yes	Yes
SoC	Depression and burn-out	Yes	No
SoC	Self-isolation due to covid-19.	Unknown	Yes

Table S2. Results from the sensitivity analysis.

	SoC (n=24)				5:2 (n=25)				LCHF (n=25)			
	Start	EoT	Delta mean, (95% CI)	P	Start	EoT	Delta mean, (95% CI)	P	Start	EoT	Delta mean, (95% CI)	P
MR-fat, mean %	14.5	10.9	-3.6 (-5.4 to -1.7)	<0.001	13.6	7.5	-6.1 (-7.8 to -4.4)	<0.001	13.7	6.5	-7.3 (-9.1 to -5.5)	<0.001
Elastography, mean kPa	7.2	5.7	-1.4 (-2.3 to -0.6)	0.001	7.2	5.5	-1.8 (-2.5 to -1.0)	<0.001	7.0	6.7	-0.3 (-1.1 to 0.5)	0.425

The main outcome adjusted for MR fat percent at baseline and sex. Delta denotes the difference in mean in absolute values from baseline to EoT. P-values are for paired t-test for change from baseline value to end of treatment. Abbreviation: EoT=end of treatment.

Table S3. Results from the per-protocol analysis

	SoC (n=20)				5:2 (n=24)				LCHF (n=20)			
	Start	EoT	Delta mean, (95% CI)	P	Start	EoT	Delta mean, (95% CI)	P	Start	EoT	Delta mean, (95% CI)	P
MR-fat, mean %	16.5	12.7	-3.8 (-6.1 to -1.6)	0.001	12.5	6.3	-6.2 (-8.2 to -4.2)	<0.001	13.8	6.1	-7.7 (-9.9 to -5.5)	<0.001
Elastography, mean kPa	7.4	6.1	-1.3 (-2.3 to -0.4)	0.007	7.6	5.8	-1.8 (-2.6 to -0.9)	<0.001	6.5	6.3	-0.2 (-1.1 to 0.8)	0.730

Delta denotes the difference in mean in absolute values from baseline to EoT. P-values are for paired t-test for change from baseline value to end of treatment.

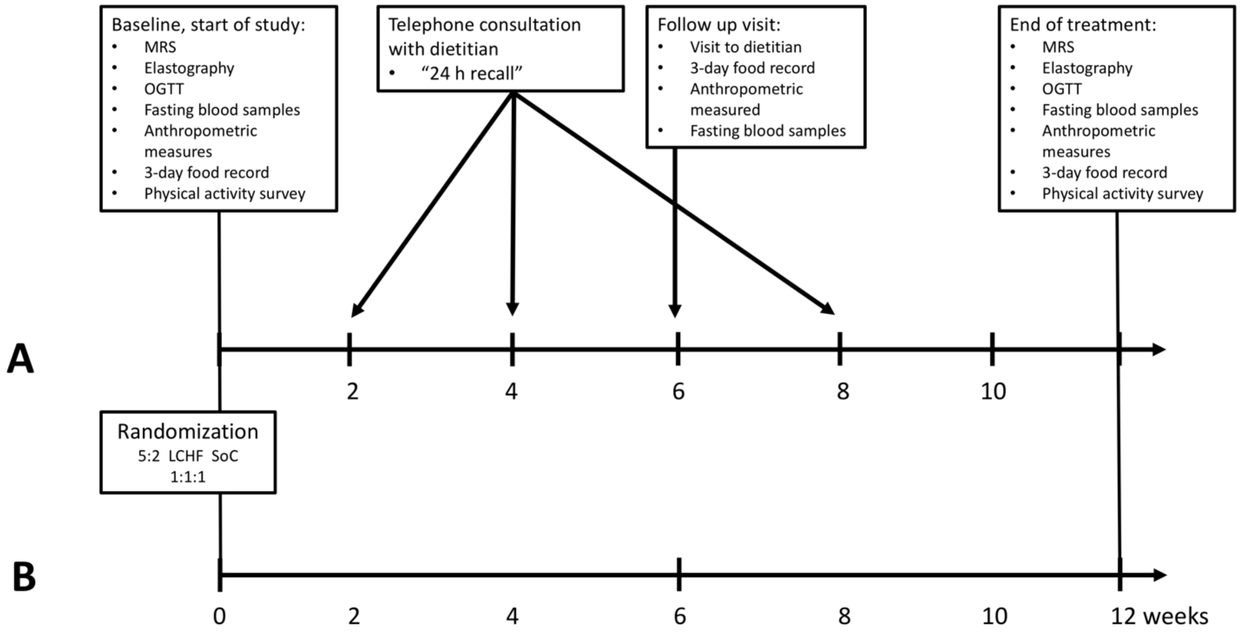

Fig. S1. Timeline describing the diet intervention. (A) the 5:2 and LCHF groups and (B) the SoC group. Abbreviations: SoC=standard-of-care. MRS=magnetic resonance spectroscopy, OGTT=oral glucose tolerance test.

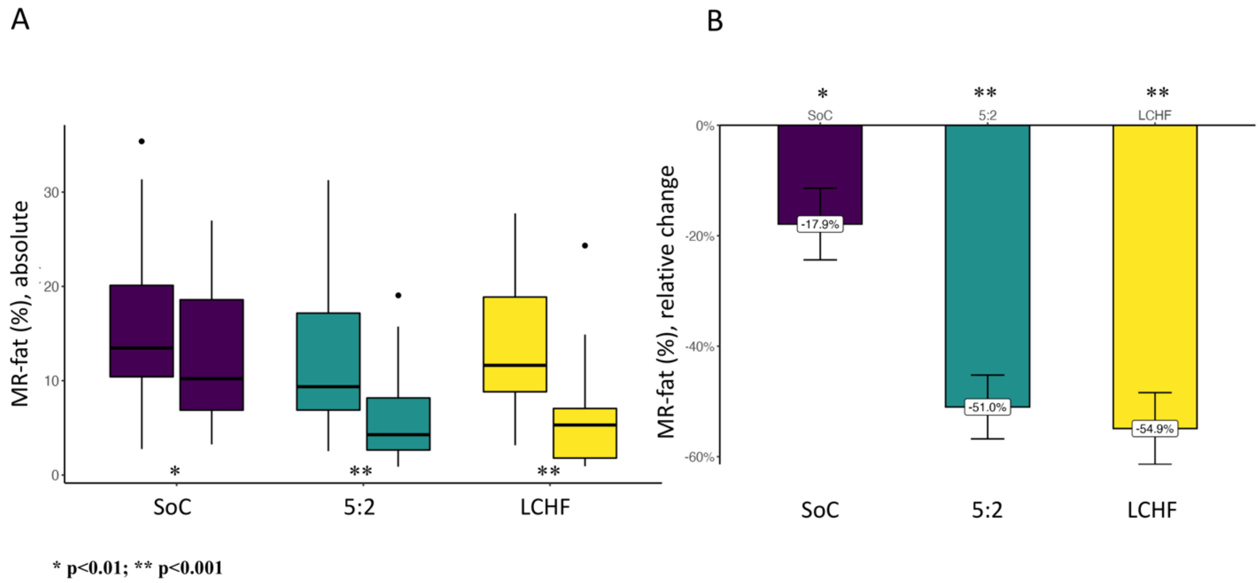

Fig. S2. Change in liver steatosis, per protocol analysis.

(A) Boxplot showing MR fat percent at baseline and at end of treatment, per group. * and ** = p-values for change within each group from baseline to end of treatment. (B) Relative change in MR fat from baseline to end of treatment, per group. MR= Magnetic resonance spectroscopy, SoC= Standard of care, 5:2= The 5:2 diet, LCHF= The low-carb high-fat diet.

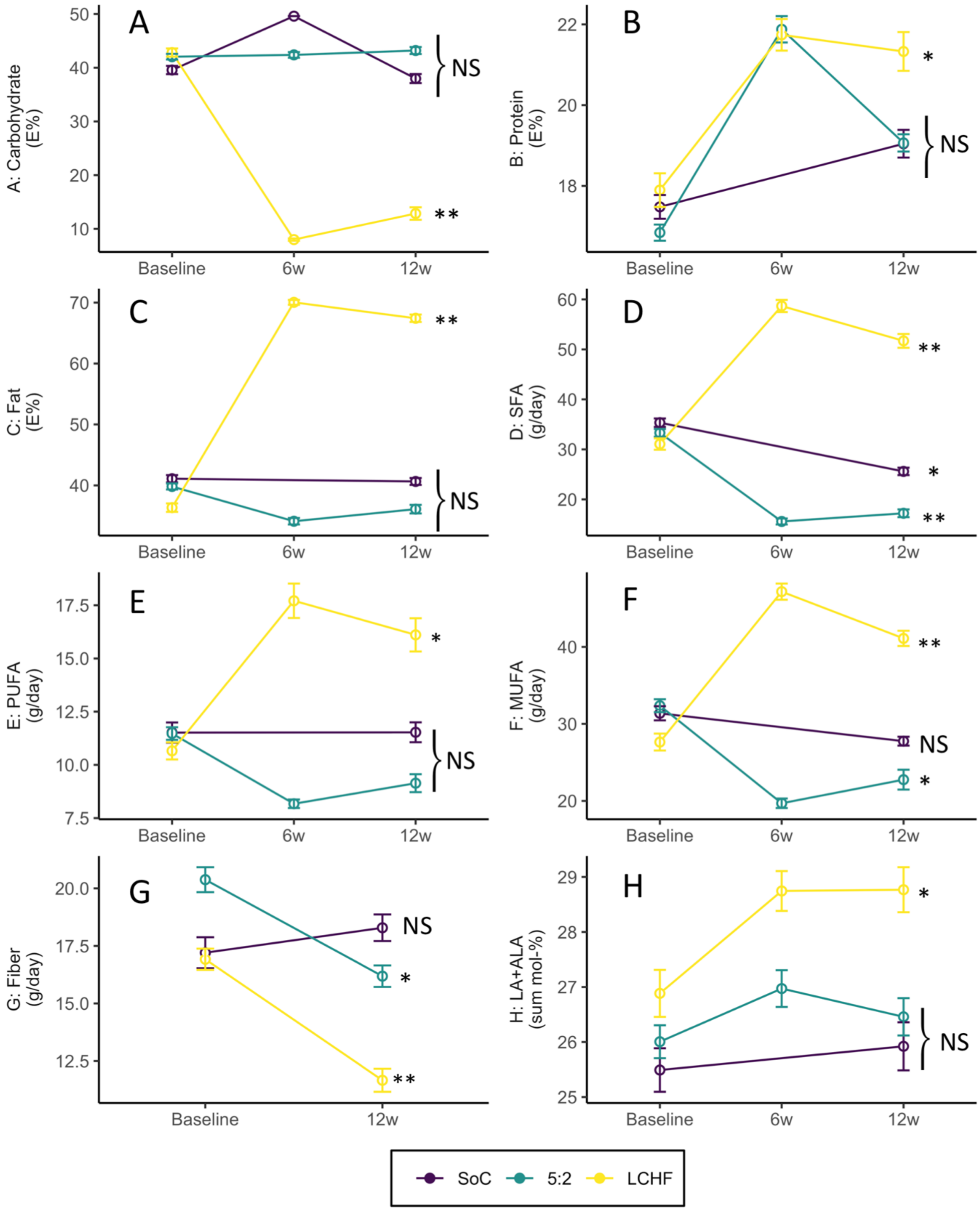

Fig. S3. Change in self-reported diet composition and plasma fatty acid levels from baseline to EoT.

Energy intake-% from (A) fat, (B) proteins, (C) carbohydrates.

Daily intake (grams/day) of (D) saturated fats, (E) monounsaturated fats, (F) polyunsaturated fats and (G) fibre.

(H) Change in the sum of relative to the total measured fatty acids of circulating linoleic acid and alpha-linoleic acid. Paired t-test: *= $p<0.05$. **=$p<0.001$. NS= not significant. Abbreviations: EoT= end of treatment, ALA=α-linolenic acid, LA=linoleic acid, SFA=saturated fatty acids, PUFA=polyunsaturated fatty acids, MUFA: monounsaturated fatty acids.

Supplementary references

1. Matthews DR, Hosker JP, Rudenski AS, Naylor BA, Treacher DF, Turner RC. Homeostasis model assessment: insulin resistance and beta-cell function from fasting plasma glucose and insulin concentrations in man. *Diabetologia.* 1985;28(7):412-419.
2. Bohn MJ, Babor TF, Kranzler HR. The Alcohol Use Disorders Identification Test (AUDIT): validation of a screening instrument for use in medical settings. *Journal of studies on alcohol.* 1995;56(4):423-432.
3. Neumann J, Beck O, Helander A, Bottcher M. Performance of PEth Compared With Other Alcohol Biomarkers in Subjects Presenting For Occupational and Pre-Employment Medical Examination. *Alcohol and alcoholism (Oxford, Oxfordshire).* 2020.
4. DABAS - Alla livsmedel på marknaden. 2020; https://www.dabas.com. Accessed 26/05/2020, 2020.
5. Folch J, Lees M, Sloane Stanley GH. A simple method for the isolation and purification of total lipides from animal tissues. *The Journal of biological chemistry.* 1957;226(1):497-509.
