# Supplementary material for: Treatment of NAFLD with intermittent calorie restriction or low-carb high-fat diet – a randomised controlled trial
Source: JHEP Rep. 2021 Feb 17;3(3):100256. doi: 10.1016/j.jhepr.2021.100256 (PMC8059083; doi:10.1016/j.jhepr.2021.100256)
Supplement: Multimedia component 2 [file mmc2.pdf]

Research program TREND study: Treatment of NAFLD using two diets

Summary

- Fatty liver (NAFLD) is the most common liver disease in the world
- Only a few patients manage to lose weight to the extent needed (>10%) to reduce the amount of fat in the liver
- It is unknown which diet method is most effective in reducing the amount of fat in the liver of patients with NAFLD
- In animal models and some studies in humans, a high-fat diet has been shown to lead to increased fat in the liver
- In this intervention study, we compare how two isocaloric diets with different fat content - periodic fasting (5: 2) and low-carbohydrate high-fat diet (LCHF) - affect the liver in patients with NAFLD

Background

Fat retention in the liver (non-fatty alcoholic liver disease, NAFLD) is the world's most common liver disease that affects approximately 15-30% of the population. Moreover, it is closely linked to obesity and insulin resistance (1,2). Brown and Goldstein have shown that high insulin production (such as insulin resistance) stimulates the production of triglycerides in the liver and the development of liver steatosis, which may be an explanation for why NAFLD is associated with diabetes mellitus type 2 and cardiovascular disease (3). Some 3-5% of patients with NAFLD develop liver cirrhosis (4). At present, there is no medical treatment for NAFLD, but the most effective method of treatment is weight loss. A weight loss of 10% or more leads to better insulin sensitivity and a reduction of fat content in the lever by nearly 100%, which reduces the risk of developing liver cirrhosis. Unfortunately, such weight loss can only be achieved in 10-20% of patients (5). There is currently no structured care for this large patient group, but usually patients are informed that they should lose weight after the diagnosis of NAFLD has been determined. How this should be done is often left to the patients themselves, which can lead to a lack of motivation and poor results. Many patients want to use one of the popular weight-loss diets, such as calorie restriction (CR) with periodic fasting (so-called 5:2 diet) or low carbohydrate high-fat diet (so-called low-carb high-fat, LCHF).

Several studies have shown that CR reduces the risk of cardiovascular disease and diabetes. Animal studies have shown that CR reduces IGF1 levels, the risk of cancer and prolongs life

(6-8). Under a long time, CR lowers blood pressure, blood sugar and blood pressure. A study has shown that a reduced incidence of atherosclerosis (atherosclerosis) is linked to CR and not to weight loss per se (9). Periodic CR, according to the so-called 5:2 diet, means that during two of the days of the week, a maximum of 25% of the daily caloric intake is recommended (i.e. about 500-600 kcal); during the other days of the week, food intake is optional, but with an emphasis on the so-called Mediterranean diet. Only a few studies have evaluated the effect of periodic CR on healthy people with overweight and patients with type-2 diabetes.

The low-carb-high-fat (LCHF) diet involves a reduction in intake of carbohydrates and a compensatory increase in fat intake. LCHF is a diet that has been widely applied in recent years, and which is popular with obese people for losing weight (13). Thus far, the scientific basis for the LCHF diet is sparse, but a similar diet (the Atkins diet) has been shown to reduce weight slightly more than comparable diets (14). In a Danish study, high-fat diets gave a more favorable lipid profile in serum than low-fat diets rich in carbohydrates (15). In animal models, however, a high-fat diet leads to increased fat retention in the liver (16, 17). The composition of fatty acids in the diet may have an impact on fat retention in the liver. Ingestion of muffins enriched with saturated fatty acids resulted in a significant increase in fat retention in the liver and visceral fat compared to the intake of muffins enriched with polyunsaturated fatty acids (18). However, in this study, patients ate more than normal, and it is unknown whether saturated fats have the same effect at concomitant calorie restriction. In one study, visceral fat and metabolic syndrome were not affected by dietary fat (19). In contrast, in other studies, dietary treatment with a high proportion of fat in the diet (55-6060 E% fat) compared to low fat in the diet (16-20 E%) has led to more pronounced fat retention in the liver (20, 21).

Thus, there are no studies that have evaluated the effects of periodic fasting or LCHF diet on fat retention in the liver, liver enzymes, liver elasticity or insulin sensitivity in patients with NAFLD.

Purpose

To investigate in a randomized, controlled study whether two common diets, the 5:2 and LCHF diet, can reduce the amount of fat in the liver, liver enzymes, liver stiffness and insulin resistance in patients with NAFLD compared to placebo (in the form of standard advice on nutritional intake). A further aim was to determine whether any of these diets have a greater effect than the other.

Primary outcome measure

- Reduction of fat in the liver, measured by MRI after 3 months of treatment with either the LCHF diet, the 5:2 diet, or placebo.

Secondary outcome measures

- Reduction of liver stiffness and liver fat as measured by elastography (FibroScan).
- Reduction of liver enzymes (ASAT, ALAT).
- Weight reduction.
- Altered body composition as measured by MRI, BIA and MAMC/TSF.
- Improvement in insulin sensitivity as measured by IR-HOMA, IGFBP-1 and Adiponectin.

Inclusion criteria

- Diagnosed NAFLD trough
    - findings of liver steatosis in radiology

- or a transaminase increase without any other known or suspected liver disease, in combination with obesity (BMI ≥30) and at least one parameter in the metabolic syndrome, and with controlled attenuation parameter (CAP)>280 in FibroScan

- Can understand Swedish to the extent necessary to comprehend the purpose and method of the study
- Signing the informed consent form

Exclusion criteria

- Diabetes mellitus type-1 or type-2 treated with insulin, GLP-1 agonists or sulfonylurea preparations (NOTE! metformin treatment is allowed)
- Other liver diseases known or detected during screening
- Other known or suspected causes of fat deposits in the liver
- Consumption of alcohol over 140 grams per week (10 standard units) for women and over 210 grams per week (15 standard units) for men
- Established liver cirrhosis in radiology, FibroScan or liver biopsy
- Chronic kidney disease stages 4 and 5 (Pt-eGFR <31ml/min)
- Pregnant and nursing women
- Implanted metal (e.g., pacemaker, aneurysm clips)
- Claustrophobia
- Participation in other clinical trials for NAFLD
- Tested any of the intervention methods within six months before screening

Method

Patients are recruited via three routes: (1) patients with known NAFLD who are followed at the liver clinic at the Gastro center; (2) patients referred to the Gastro center from primary care for investigation of suspected NAFLD; and (3) by an advertisement in the local press

(see Appendix 1). Patients are called to a doctor's appointment at the liver clinic where they are informed about the study orally and in writing.

Patients who meet the inclusion criteria and sign the informed consent form are screened for any exclusion criteria. After screening and inclusion, baseline tests are performed (specified below). A total of 75 patients, balanced for sex, will be randomized to either treatment with the LCHF diet, 5:2 diet, or placebo (see below). The diet intervention takes place in collaboration with Kerstin Brismar's research group at Karolinska. Solna by dietitians and doctors. Methodology and infrastructure have been developed through other diet intervention studies, with logistics that have been tested on >100 patients with and without type2 diabetes. At both the start and conclusion of the study, the liver's fat content is checked with an MRI and liver stiffness with elastography. Differences in fat content and liver stiffness will be statistically analyzed using the rank-sum-test primarily according to the intention-to-treat principle as well as in sensitivity analysis by the per-protocol principle.

Because there are no data on the effect of diets on the reduction of liver fat, it is difficult to perform a power analysis. If data are extrapolated from other weight loss interventions where liver fat data are available and assume that dietary intervention groups will lose approximately 5% of their weight over three months, this represents approximately 30% reduction in liver fat. Assuming that the placebo group reduces the amount of fat in the liver by 10%, and with a standard deviation of 20%, at least 16 people in each arm will be needed to see differences between the groups with 80% power and an alpha value of 0.05. Because some loss of participants can be expected to occur, we want to include 25 people in each arm.

Screening examination

During screening, the following will take place:

- Endorsement of informed consent
- Medical history, heredity, drug use, weight history
- Status (heart, lung, abdominal status, sitting blood pressure and heart rate), height, weight, waist circumference, hip measurements
- Routine blood test analysis (Sentto Kemlab): HbA1c, blood status, creatine, ALT, ALP, ferritin, glucose, hsCRP and TSH, Pt-eGFR (Cystatin C)
- Supplementing liver investigation if not previously performed (hepatitis serology, autoantibodies, protein fractions)

Baseline samples

If a patient meets the inclusion criteria and has no post-screening exclusion criteria, the following baseline tests will be performed after a 8-hour overnight fast. (*Participants should not have fasted the day before taking blood tests.)* The examinations are done at the Hepatological Unit, Huddinge and the Radiology Clinic, Huddinge. A total of 120 ml of blood is taken at baseline.

- Triglycerides, total cholesterol, LDL cholesterol, HDL cholesterol, AST, ALT, ALP, GT, insulin, glucose, C-peptide, ApoB/ApoA1 ratio, TSH (sent to Kemlab)
- Urine test: ketones in urine (checked with urine strips at reception).
- Research blood samples (serum, heparin plasma and EDTA plasma), which are stored after centrifugation as noted below in cryotubes (0.5 ml/tube) and frozen for later analysis for the following parameters: markers of vascular inflammation and oxidative stress, GH, IGF-I, IGFBP-1, IGFBP-2, leptin, adiponectin, CK-18 fragments and fatty acid composition in plasma (e.g., for validation of the diet diary). The samples are saved as serum (7 x 0.5 ml), EDTA plasma (3 x 0.5 ml) and heparin plasma (4 x 0.5 ml) in cryotubes with 0.5 ml/tube at -70°C.

- Urine sample: 8 ml of morning urine is frozen at -70°C for later analyses of parameters reflecting oxidative stress and metabolomics.
- Feces test: The participant receives a stool test kit with sampling instructions at the screening visit. The sample will be included in the baseline examination. The sample is saved at -70°C for later analysis of the composition of microbiota and metabolites (see Appendix 2). The composition of the microbiota is analyzed using DNA technology (either that the 16SrRNA gene is amplified and sequenced or metagenomics in which the entire bacterial genome is sequenced).
- Whole blood for DNA analysis for the determination of polymorphisms related to NAFLD (PNPLA3, TM6SF2, et al.) (7 ml). Saved at -70°C.
- Elastography with FibroScan to measure liver stiffness and measurement of fat content with CAP.
- MRI scans of the liver to measure fat content (including body composition and fat distribution) will be performed at the MRI unit, Karolinska Huddinge. The measurement is done without a contrast agent and takes about 45 minutes. During the measurement, a short-term vibration wave is sent in from the right flank to measure liver stiffness. In one-third of the patients, methodological control of reproducibility will be carried out by conducting 2 MRI examinations at the same occasion.
- Bioimpedance measurement via the arms and legs (measurement of body composition, non-invasive and risk-free).
- Upper arm muscle circumference and triceps skin folds will be measured with a measuring tape and calipers.
- Oral glucose tolerance test – (OGTT).
- 3-day diet diary will be applied to compare the patient's food intake before the start of the study and after the intervention. The diet diary will be validated by blood tests to analyze fatty acid composition in plasma.

- Participants will be asked to complete validated internet-based questionnaires on physical activity level (Active-Q©), eating habits (Meal-Q©), alcohol habits (AUDIT), Consolation Eating Survey, Ladder of Life.

Randomization and dietary treatment

- After baseline samples, patients will be randomized 1:1:1 to the LCHF, 5:2, or placebo diet (meaning standard lifestyle advice according to clinical practice, see Appendix 3). The study is planned to last for 3 years, so that every three to six months, 5-10 participants per group are randomized. Each group will be randomized to one of the respective diets. Group information will be given about diet at a meeting for each group, at which time menus will be distributed.
- **The 5:2 diet** for women means that for 2 days a week, they eat 25% (= 500 kcal of intake of 2000 kcal/day) and for the other 5 days of the week, eat the full 2000 kcal/day. Hence, a total of 11,000 kcal/week, which, on average, gives about 1,556 kcal/day. For men, 600 kcal/day are consumed during the fasting days, calculated on a 2,400-kcal daily intake, resulting in a weekly intake of 13,200 kcal =1,886 kcal/day. For variation, each participant has energy-calculated menus to choose from for breakfast, lunch and dinner during the "fasting days". Information about the Nordic nutritional recommendations will be given as an alternative on the 5 days when the participants eat freely. They can decide for themselves two or three meals per day, choosing from four alternatives for each meal. Participants may vary between the selected menus during the days of fasting. During the study, the participants will be instructed to record in a diary which menu they choose and which day of the week and whether they follow the menu or not. The diary will be distributed during the information meeting and the participants will take it with them at the return visit.

- **The LCHF diet** implies a restriction on carbohydrate intake to a maximum of 10% of the total energy intake. Approximate energy distribution will be about 5-10E% carbohydrates, 50-80 E% fat and 15-40 E% protein. The group is instructed to maintain a diet with approximately the same calorie intake as the 5: 2 distribution over the week, i.e. an average of about 1,600 kcal/day for women and 1,900 kcal/day for men. The diet is based on the intake of meat, fish, eggs, surface vegetables, vegetable oils and dairy fats (e.g., butter, cream, cheese). Minimized will be the intake of sugary drinks, juice, sugar, bread, pasta, rice, pie, potatoes and other carbohydrate-rich foods, including certain fruits.
- **The placebo diet** means that patients are informed about the diet by their doctor at the clinic in accordance with clinical practice. They are not informed about a specific diet, or group information or dietician contact. The dietary advice includes, among other things, a reduction in the intake of sweets, avoidance of excessive portions, eating three meals a day regularly and do not drink more than a maximum of 10 standard glasses of alcohol (one standard glass corresponds to 12-14 grams of pure alcohol) per week (see Appendix 3).

Start of treatment

Treatment takes place separately for the 5:2 and LCHF groups in connection with visits to the endocrine clinic, Solna. The placebo group receives no intervention.

- Participants are given group information about diet and menu suggestions led by a dietitian and doctor at the endocrine unit in Solna. At the meeting, participants are informed in groups about the 5:2 method and the LCHF diet, depending on randomization (different groups at different times): Approximately 5-8 participants per group per occasion.

Phone follow-up with a dietician (5:2 and LCHF)

Follow-up occurs after 2, 4 and 8 weeks of treatment. The dietician encourages continued dietary treatment and records adherence to diet.

Examinations after 6 weeks

After 6 weeks, the patients will be called to a dietician and research nurse to undergo the following examinations:

- Weight control, abdominal range, hip circumference
- The participants will be asked to leave their dietary diaries (filled in for 3 days during the week before the 6-week control)
- Bioimpedance measurement
- Upper arm muscle circumference and triceps skinfolds measured with a measuring tape and calipers
- Complementary dietary information and menu suggestions
- Survey on physical activity level, alcohol habits (AUDIT), Comfort Eater survey, Ladder of Life, as well as questions about difficulties in adhering to the diet.
- Blood tests (liver status, blood lipids) and samples for fatty acid composition in plasma (e.g., to validate the dietary diary). In addition, serum and plasma samples for research will be taken in the same way as at baseline. In total, 90 ml of blood will be taken at the 6-week control.

Investigations at the end of the intervention (at 3 months)

After 3 months, the following examinations are repeated after 8 hours of fasting. (*Participants should not have fasted the day before taking blood.)* A total of 110ml of blood will be taken at the end of the study.

- HbA1c, blood status, creatine, ASAT, ALAT, ALP, GT, ferritin, glucose, triglycerides, total cholesterol, LDL cholesterol, HDL cholesterol, insulin, glucose, C-peptide, ApoB/ApoA1 ratio, hsCRP, HbA1c (sent to Kemlab)
- Urine test: ketones in urine (checked with urine strips at reception)
- Research blood samples (serum, heparin plasma and EDTA plasma), which are stored after centrifugation as noted below in cryotubes (0.5 ml/tubes), are taken for analysis of the following parameters: markers of vascular inflammation and oxidative stress for GH (growth hormone), IGF-I, IGFBP-1, IGFBP-2, leptin, adiponectin, cortisol, prolactin, sex hormones (free testosterone and free estradiol), apoptosis (CK-18 fragments) and samples for fatty acid composition in plasma (validation of the dietary diary). The samples will be saved as serum (7 x 0.5 ml), EDTA plasma (3 x 0.5 ml) and heparin plasma (4 x 0.5 ml) in cryotubes with 0.5 ml/tube at -70°C until later analyses.
- Urine tests: morning urine, 8 ml will be saved at -70°C for later analyses of parameters reflecting oxidative stress and metabolomics.
- Feces test: The participants will receive a stool test kit with sampling instructions at the screening visit. The samples will be taken at the examination. The sample will be saved at -70°C for later analysis of the composition of microbiota and metabolites.
- Elastography with FibroScan to measure liver stiffness, as well as measurement of fat content CAP.
- MRI scan with H1-MRS to measure total fat content in the liver will be performed at the MRI unit, Karolinska, Huddinge
- Weight control, abdominal range, hip circumference
- Upper arm muscle circumference and triceps skin folds will be measured with a measuring tape and calipers
- The participants will be asked to leave their dietary diaries (filled in for 3 days during the week before the 6-week control)

- The participants will be asked to leave their dietary diaries (filled in for 3 days in the week before the 3-month control, including registration of selected menus during the "fasting days")
- Bioimpedance measurement
- OGTT
- Survey on physical activity level (Active-Q©),eating habits (Meal-Q©),) alcohol habits (AUDIT), Consolation Eater survey, Ladder of Life and difficulties in following the diet.

Follow-up

To study the long-term effects of the treatment, patients will be asked to take part in further follow-ups after 6 and 12 months for bioimpedance, weight, waist circumference, blood tests (same as in the 3-month follow-up) and questionnaires, as well as an MRI scan after 12 months.

Patient benefits

Fatty liver disease is the most common liver disease in the world, and a subgroup of these patients is at risk of developing severe complications. Being able to advise these patients on which diet is most effective in reducing the amount of fat in the liver can eventually affect liver-related morbidity and ultimately contribute to a reduction in overall mortality in this patient group. It can also contribute to a better quality of life if a more structured and equality-based treatment can be offered to patients.

Risks

There is a potential risk that the LCHF diet may lead to an increase in fat retention in the liver. However, given the concomitant calorie restriction, the risk is considered minimal. At the 6-week control, fat retention will be evaluated through blood tests (control of triglycerides

and liver enzymes). If ALAT, ASAT, ALP, or bilirubin rises >3 times baseline levels, diet treatment will be discontinued.

Preliminary data

All logistics, including research nurses and nutritionists, as well as infrastructure for handling responses to online questionnaires and blood samples, will be set up at each unit (Hepathologist Karolinska, Radiology Department. Huddinge and Endocrinologist Solna).

References

1. Loomba R, Sanyal AJ. The global NAFLD epidemic. Nature reviews Gastroenterology & hepatology. 2013 Nov;10(11):686-90.
2. Younossi ZM, Koenig AB, Abdelatif D, Fazel Y, Henry L, Wymer M. Global epidemiology of nonalcoholic fatty liver disease-Meta-analytic assessment of prevalence, incidence, and outcomes. Hepatology. 2016 Jul;64(1):73-84.
3. Owen JL, Zhang Y, Bae SH, Farooqi MS, Liang G, Hammer RE, et al. Insulin stimulation of SREBP-1c processing in transgenic rat hepatocytes requires p70 S6-kinase. Proceedings of the National Academy of Sciences of the United States of America. [Research Support, NIH, Extramural
Research Support, Non-U.S. Gov't]. 2012 Oct 02;109(40):16184-9.
4. Anstee QM, Targher G, Day CP. Progression of NAFLD to diabetes mellitus, cardiovascular disease or cirrhosis. Nature reviews Gastroenterology & hepatology. 2013 Jun;10(6):330-44.
5. Vilar-Gomez E, Martinez-Perez Y, Calzadilla-Bertot L, Torres-Gonzalez A, Gra-Oramas B, Gonzalez-Fabian L, et al. Weight Loss Through Lifestyle Modification Significantly Reduces Features of Nonalcoholic Steatohepatitis. Gastroenterology. 2015 Aug;149(2):367-78.e5; quiz e14-5.
6. Colman RJ, Anderson RM, Johnson SC, Kastman EK, Kosmatka KJ, Beasley TM, et al. Caloric restriction delays disease onset and mortality in rhesus monkeys. Science (New York, NY). 2009 Jul 10;325(5937):201-4.
7. Mattison JA, Roth GS, Beasley TM, Tilmont EM, Handy AM, Herbert RL, et al. Impact of caloric restriction on health and survival in rhesus monkeys from the NIA study. Nature. 2012 Sep 13;489(7415):318-21.
8. Meydani M, Das S, Band M, Epstein S, Roberts S. The effect of caloric restriction and glycemic load on measures of oxidative stress and antioxidants in humans: results from the CALERIE Trial of Human Caloric Restriction. The journal of nutrition, health & aging. 2011 Jun;15(6):456-60.
9. Ahmet I, Tae HJ, de Cabo R, Lakatta EG, Talan MI. Effects of calorie restriction on cardioprotection and cardiovascular health. Journal of molecular and cellular cardiology. 2011 Aug;51(2):263-71.
10. Brown JE, Mosley M, Aldred S. Intermittent fasting: a dietary intervention for prevention of diabetes and cardiovascular disease. Br J Diabetes Vasc Dis. 2013;13(2):68-72.
11. Heilbronn LK, Ravussin E. Calorie restriction and aging: review of the literature and implications for studies in humans. The American journal of clinical nutrition. 2003 Sep;78(3):361-9.

12. Varady KA, Hellerstein MK. Alternate-day fasting and chronic disease prevention: a review of human and animal trials. The American journal of clinical nutrition. 2007 Jul;86(1):7-13.
13. Johansson I, Nilsson LM, Stegmayr B, Boman K, Hallmans G, Winkvist A. Associations among 25-year trends in diet, cholesterol and BMI from 140,000 observations in men and women in Northern Sweden. Nutrition journal. 2012;11:40.
14. Gardner CD, Kiazand A, Alhassan S, Kim S, Stafford RS, Balise RR, et al. Comparison of the Atkins, Zone, Ornish, and LEARN diets for change in weight and related risk factors among overweight premenopausal women: the A TO Z Weight Loss Study: a randomized trial. Jama. 2007 Mar 7;297(9):969-77.
15. Thorning TK, Raziani F, Bendsen NT, Astrup A, Tholstrup T, Raben A. Diets with high-fat cheese, high-fat meat, or carbohydrate on cardiovascular risk markers in overweight postmenopausal women: a randomized crossover trial. The American journal of clinical nutrition. 2015 Sep;102(3):573-81.
16. Eccleston HB, Andringa KK, Betancourt AM, King AL, Mantena SK, Swain TM, et al. Chronic exposure to a high-fat diet induces hepatic steatosis, impairs nitric oxide bioavailability, and modifies the mitochondrial proteome in mice. Antioxidants & redox signaling. 2011 Jul 15;15(2):447-59.
17. Wang XH, Li CY, Muhammad I, Zhang XY. Fatty acid composition in serum correlates with that in the liver and non-alcoholic fatty liver disease activity scores in mice fed a high-fat diet. Environmental toxicology and pharmacology. 2016 Apr 30;44:140-50.
18. Rosqvist F, Iggman D, Kullberg J, Cedernaes J, Johansson HE, Larsson A, et al. Overfeeding polyunsaturated and saturated fat causes distinct effects on liver and visceral fat accumulation in humans. Diabetes. 2014 Jul;63(7):2356-68.
19. Veum VL, Laupsa-Borge J, Eng O, Rostrup E, Larsen TH, Nordrehaug JE, et al. Visceral adiposity and metabolic syndrome after very high-fat and low-fat isocaloric diets: a randomized controlled trial. The American journal of clinical nutrition. 2016 Nov 30.
20. van Herpen NA, Schrauwen-Hinderling VB, Schaart G, Mensink RP, Schrauwen P. Three weeks on a high-fat diet increases intrahepatic lipid accumulation and decreases metabolic flexibility in healthy overweight men. J Clin Endocrinol Metab. [Randomized Controlled Trial
Research Support, Non-U.S. Gov't]. 2011 Apr;96(4):E691-5.
21. Westerbacka J, Lammi K, Hakkinen AM, Rissanen A, Salminen I, Aro A, et al. Dietary fat content modifies liver fat in overweight nondiabetic subjects. J Clin Endocrinol Metab. [Research Support, Non-U.S. Gov't]. 2005 May;90(5):2804-9.
