# Supplementary material for: Treatment of NAFLD with intermittent calorie restriction or low-carb high-fat diet – a randomised controlled trial
Source: JHEP Rep. 2021 Feb 17;3(3):100256. doi: 10.1016/j.jhepr.2021.100256 (PMC8059083; doi:10.1016/j.jhepr.2021.100256)
Supplement: Multimedia component 3 [file mmc3.pdf]

JHEP|Reports

JHEP Reports

CTAT methods

Tables for a “Complete, Transparent, Accurate and Timely account” (CTAT) are now mandatory for all revised submissions. The aim is to enhance the reproducibility of methods.

- Only include the parts relevant to your study
- Refer to the CTAT in the main text as ‘Supplementary CTAT Table’
- Do not add subheadings
- Add as many rows as needed to include all information
- Only include one item per row

If the CTAT form is not relevant to your study, please outline the reasons why:

1.1 Antibodies

Name	Citation	Supplier	Cat no.	Clone no.

1.2 Cell lines

Name	Citation	Supplier	Cat no.	Passage no.	Authentication test method

1.3 Organisms

Name	Citation	Supplier	Strain	Sex	Age	Overall n number

1.4 Sequence based reagents

Name	Sequence	Supplier

1.5 Biological samples

Description	Source	Identifier

1.6 Deposited data

Name of repository	Identifier	Link

Created : November, 2018

1.7 Software

Software name	Manufacturer	Version
R		3.6.1

1.8 Other (*e.g.* drugs, proteins, vectors etc.)

1.9 Please provide the details of the corresponding methods author for the manuscript:

Magnus Holmer
Department of Liver Diseases, Division of Upper GI-diseases
C1:77 Karolinska University Hospital Huddinge
141 86 Stockholm
Telephone: +46 (0)8 58581805 | +46 (0) 709508255
Fax +46 (0)8 - 585 823 35
Email: magnus.holmer@ki.se

2.0 Please confirm for randomised controlled trials all versions of the clinical protocol are included in the submission. These will be published online as supplementary information.

confirmed
